# Supplementary material for: Multitask learning of a biophysically-detailed neuron model
Source: PLoS Comput Biol. 2024 Jul 31;20(7):e1011728. doi: 10.1371/journal.pcbi.1011728 (PMC11318869; doi:10.1371/journal.pcbi.1011728)
Supplement: S1 File — (PDF) [file pcbi.1011728.s001.pdf]

1 Supporting Information

1.1 Measuring Diversity in MMoE and MMoEEx

In principle, MTL problems are expected to benefit from higher representational diversity, by which we mean that the representations provided by the different experts capture different aspects of the input data. At least in theory, the MMoEEx architecture was constructed to promote higher diversity among representations, however as mentioned before, we did not observe any clear relationship between diversity and improved performance as measured by the expert diversity metrics discussed in the Methods and Materials section. Moreover, after retraining our models we observed that none of the diversity metrics were consistent between runs, see figure 1. For context we show the training and validation loss of the three models in Figure 2. Also, note that all three metrics follow the same pattern within a training session. This result supports the point of view that, at least for the task at hand, a large amount of diversity is not required to find a solution.

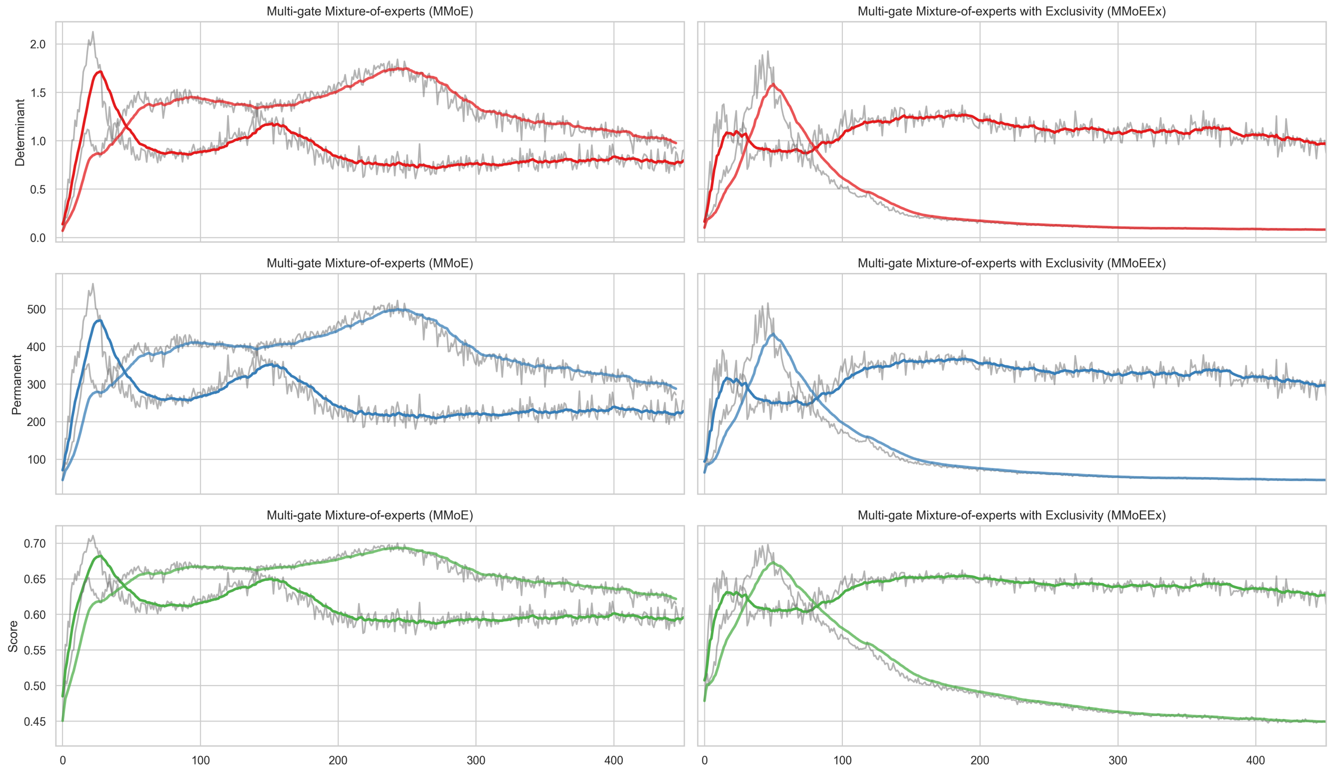

Figure 1: Smoothed time series plot of the evolution of the three diversity metrics (determinant, permanent and score) for two different training runs for both the MMoE (left) and the MMoEEx models (right).

1.2 Loss-Balanced Task Weighing for MMoE and MMoEEx

Despite the overall effectiveness of soft parameter models in achieving high average task performance, it is important to acknowledge the potential for negative transfer learning which could lead to a multi-task model under-performing on specific tasks. To address this issue, Loss-Balanced Task Weighting (LBTW) was proposed in the literature as a valuable tool for mitigating potential negative transfer effects. LBTW applies a dynamic update to the task weights in the loss function used during model training. Detailed information regarding the specific loss function and weight updates used by LBTW can be found in the Methods and Materials section. Here, we present results obtained by applying LBTW to the MMoE and MMoEEx architectures, and compare them to the previously described training and generalisation performance of the MMoE and MMoEEx architectures without task-balancing.

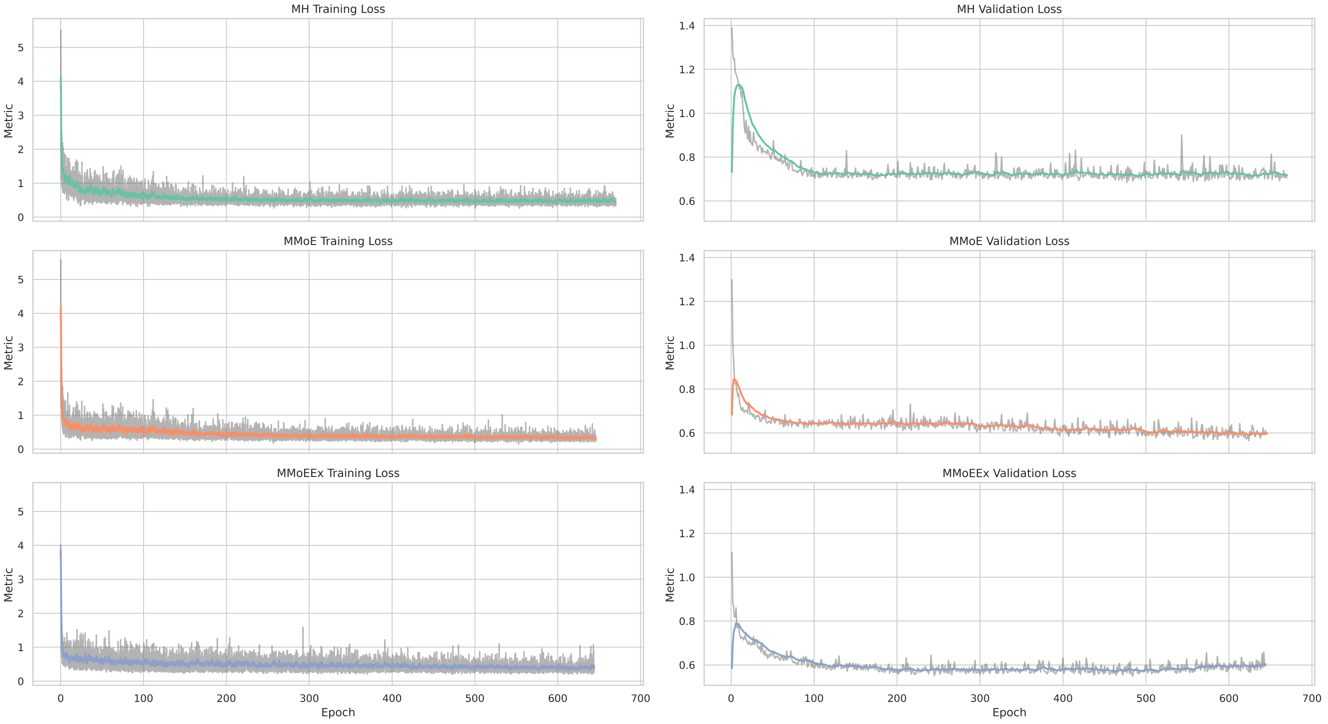

Figure 2: Smoothed time series plot of the evolution of the training and validation loss for all three models.

The hard parameter model trained with LBTW achieved a minimum validation loss of 0.718, which is higher than the loss of 0.680 obtained through the standard training procedure. Because LBTW was originally designed for soft parameter sharing models only, the lack of improvement in this case was not unexpected. However, the LBTW-trained MMoE and MMoEEx models unfortunately also exhibited higher validation losses compared to the standard training procedures. More specifically, the LBTW-trained MMoE model achieved a validation loss of 0.626, while the standard training procedure resulted in a loss of 0.533. Similarly, the LBTW-trained MMoEEx model had a validation loss of 0.634, whereas the standard training procedure yielded a lower validation loss of 0.551. For a visual representation of the individual validation losses at the end of the training run, refer to Figure 3.

1.3 Calculation of Extracellular Potentials

The membrane currents passing through cellular membranes in the vicinity of an electrode give rise to measurable extracellular potentials. One of the downstream applications of the distilled biophysically-detailed models we have presented in this paper is predicting such extracellular potentials, as was shown in Figure **??** of the main text. In this supplementary information we discuss the calculation of the extracellular potential based on the prediction of our multi-task learned models in more detail. To calculate them we rely on volume conductor theory [1], which dictates that assuming an infinite homogeneous and isotropic extracellular medium, the potential ϕ resulting from a multi-compartment neuron model is given by,

$$V_e(\mathbf{r_e}, t) = \frac{1}{4\pi\sigma} \sum_{n=0}^{N} \frac{I_n(t)}{|\mathbf{r_e} - \mathbf{r_n}|} \,. \tag{1}$$

In this equation [2], $I_n(t)$ denotes the transmembrane currents in a compartment positioned at $\mathbf{r_n}$, and $\mathbf{r_e}$ indicates the the position of the electrode. The sum runs over all N compartments of the biophysically-detailed neuron model, and σ is the extracellular conductivity which is experimentally determined to be around $0.3Sm^{-1}$ for cortical grey matter [3]. The multi-task learning models predicted membrane potentials, and not membrane currents, but given full knowledge of both the membrane potentials and the cell model itself, the corresponding membrane currents could be directly

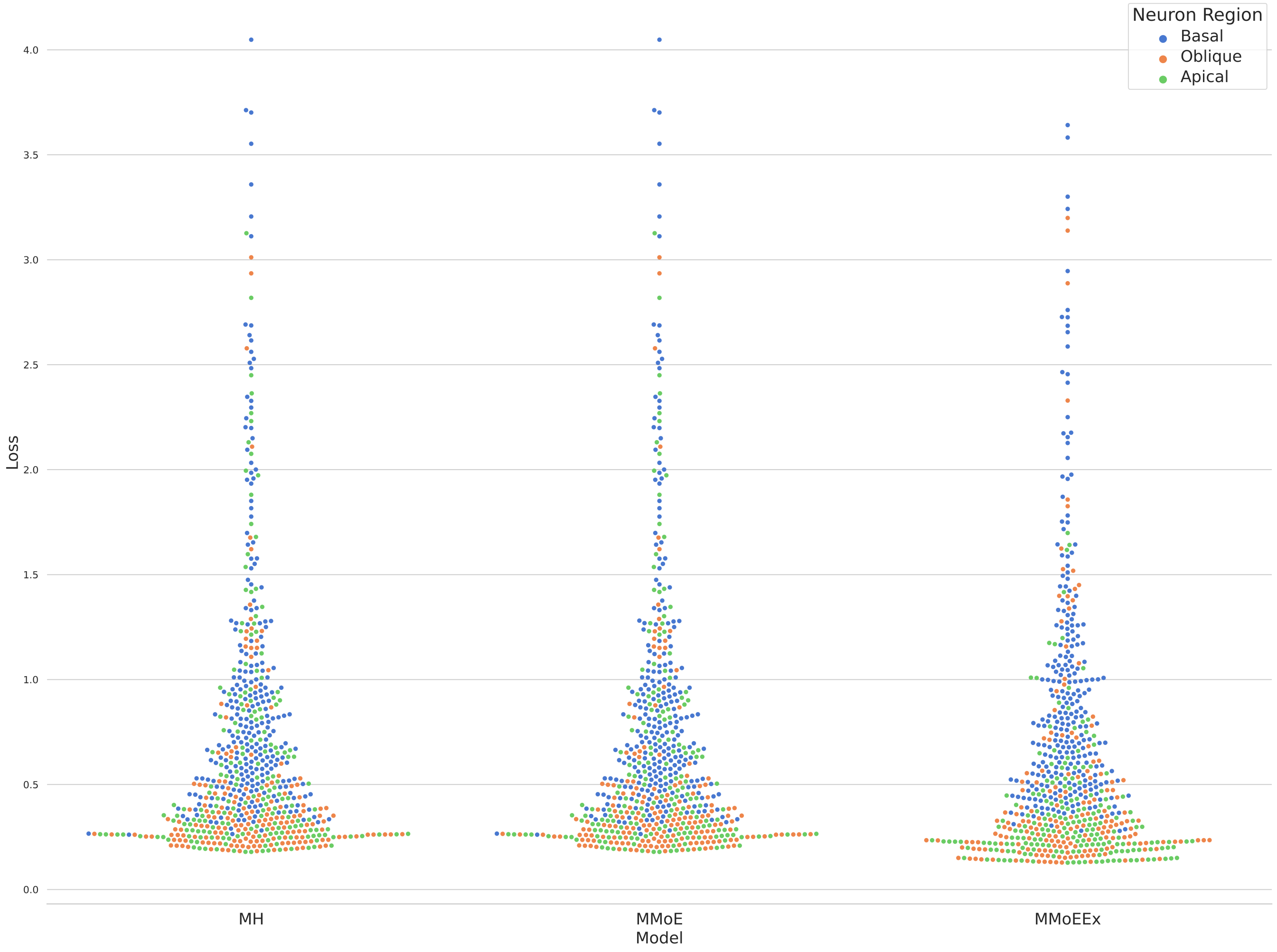

Figure 3: Swarm plots of the validation loss for each of the compartments (basal, oblique, apical) of the three LBTW-trained models: Hard Parameter Sharing (MH), Multi-gate Mixture-of-experts (MMoE), and Multi-gate Mixture-of-Experts with Exclusivity (MMoEEx).

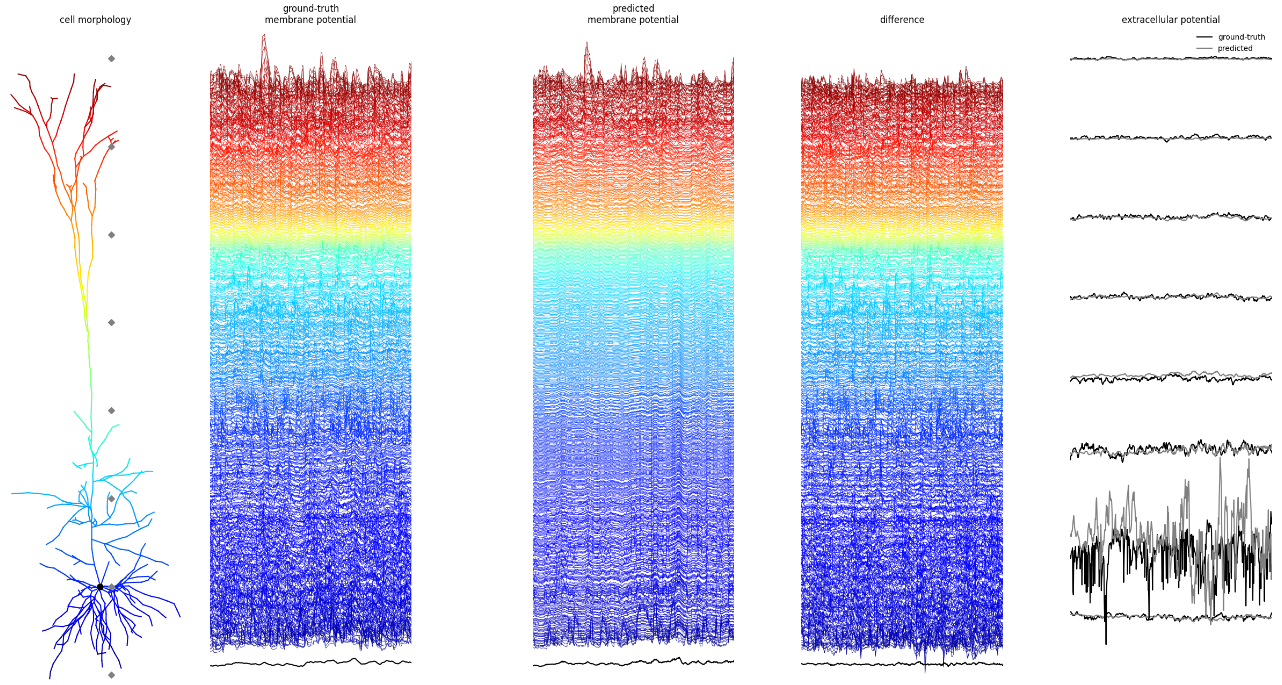

Figure 4: From left to right. Illustration of a biophysically-detailed model of a multi-compartment cortical layer V pyramidal cell model. Membrane voltages as calculated by a biophysically-detailed simulations of the multi-compartment model, used as the ground truth throughout this paper. Membrane voltages as predicted by our best-performing multi-task learning architecture, one time step at a time (1 ms). Comparison between the ground truth and predicted extracellular potentials calculated at eight points representing the position of the electrodes.

inferred (see the LFPy documentation regarding the method "get_transformation_matrix_vmem_to_imem"). Using the inferred membrane currents and the software package LFPy [4], we calculated the resulting extracellular potentials.

Extracellular potentials are a compound result of the contributions of different neuron compartments, and due to the fact that we do not explicitly train our models to predict them, accurate prediction of extracellular potentials is not necessarily a given. While on average our predictions are about as accurate as the experimental variance of the Hay model, some compartments or specific patterns of neural activity may be more or less accurately predicted. For instance as shown in Figure 4, the predictions of membrane voltages in apical and oblique compartments match the ground truth closely, whereas predictions for basal compartments are generally less accurate. As a result of this spatial variance, extracellular potentials near the soma are less accurately predicted in this specific case. Therefore, the use of these distilled biophysically-detailed neuron models in downstream prediction of extracellular potential should be handled with care.

For simulations driven by an external population the LFP can be predicted directly using the methods discussed in this section [2]. However when internal connectivity is involved, the application of the methods from this paper require an additional preprocessing step. As can be seen in the examples shown in the paper, the model is capable of reproducing spiking behavior without having explicitly learned the binary somatic spike prediction task. To predict LFPs, in the case of network simulations with internal connectivity, we advise to set a threshold on the voltage in the soma in each neuron and use that to determine whether or not there is an outgoing spike from that neuron.

References

1. P. L. Nunez and R. Srinivasan, *Electric fields of the brain: the neurophysics of EEG*. Oxford University Press, USA, 2006.

2. T. V. Ness, G. Halnes, S. Næss, K. H. Pettersen, and G. T. Einevoll, “Computing extracellular electric potentials from neuronal simulations,” in *Computational Modelling of the Brain: Modelling Approaches to Cells, Circuits and Networks*, pp. 179–199, Springer, 2021.

3. G. T. Einevoll, C. Kayser, N. K. Logothetis, and S. Panzeri, “Modelling and analysis of local field potentials for studying the function of cortical circuits,” *Nature Reviews Neuroscience*, vol. 14, no. 11, pp. 770–785, 2013.

4. E. Hagen, S. Næss, T. V. Ness, and G. T. Einevoll, “Multimodal modeling of neural network activity: computing lfp, ecog, eeg, and meg signals with lfpy 2.0,” *Frontiers in neuroinformatics*, vol. 12, p. 92, 2018.
