# Supplementary figures and images for: Multitask learning of a biophysically-detailed neuron model
Source: PLoS Comput Biol. 2024 Jul 31;20(7):e1011728. doi: 10.1371/journal.pcbi.1011728 (PMC11318869; doi:10.1371/journal.pcbi.1011728)

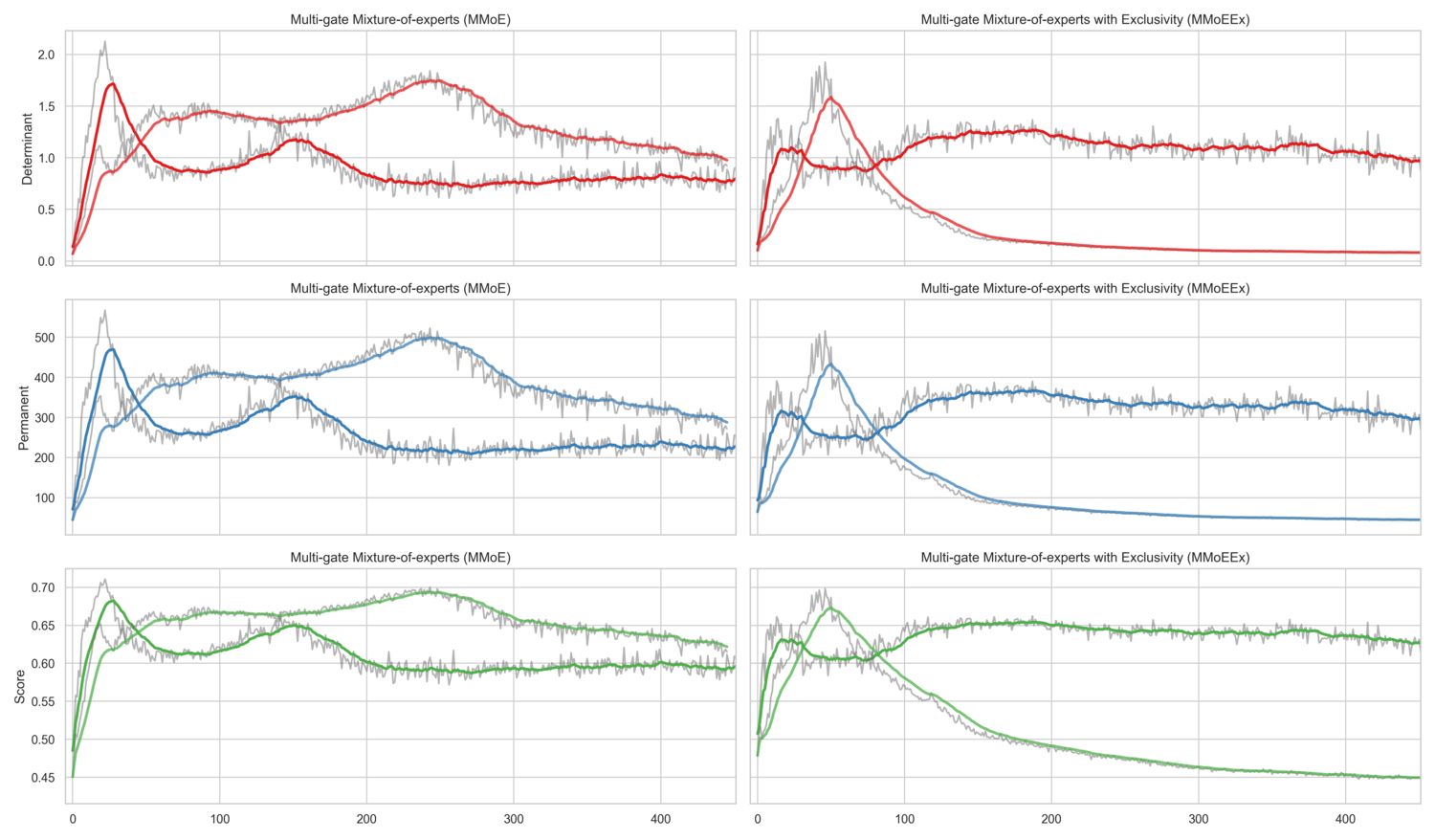

Supplement: S1 Fig — (TIFF) [file pcbi.1011728.s002.tiff]

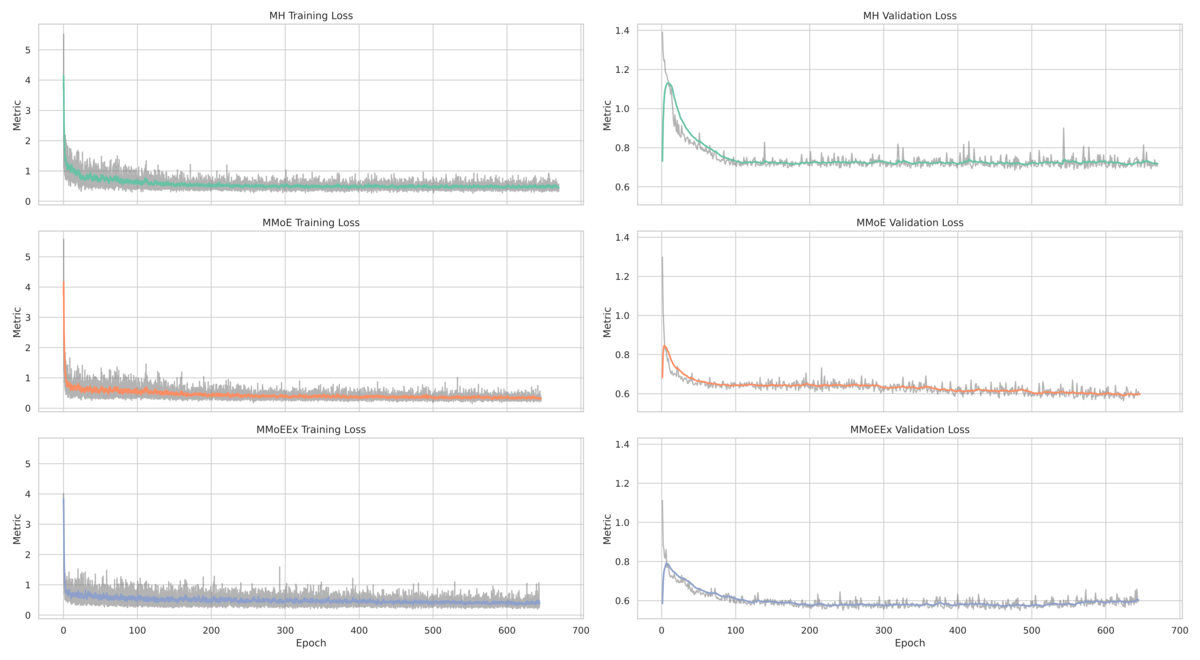

Supplement: S2 Fig — (TIFF) [file pcbi.1011728.s003.tiff]

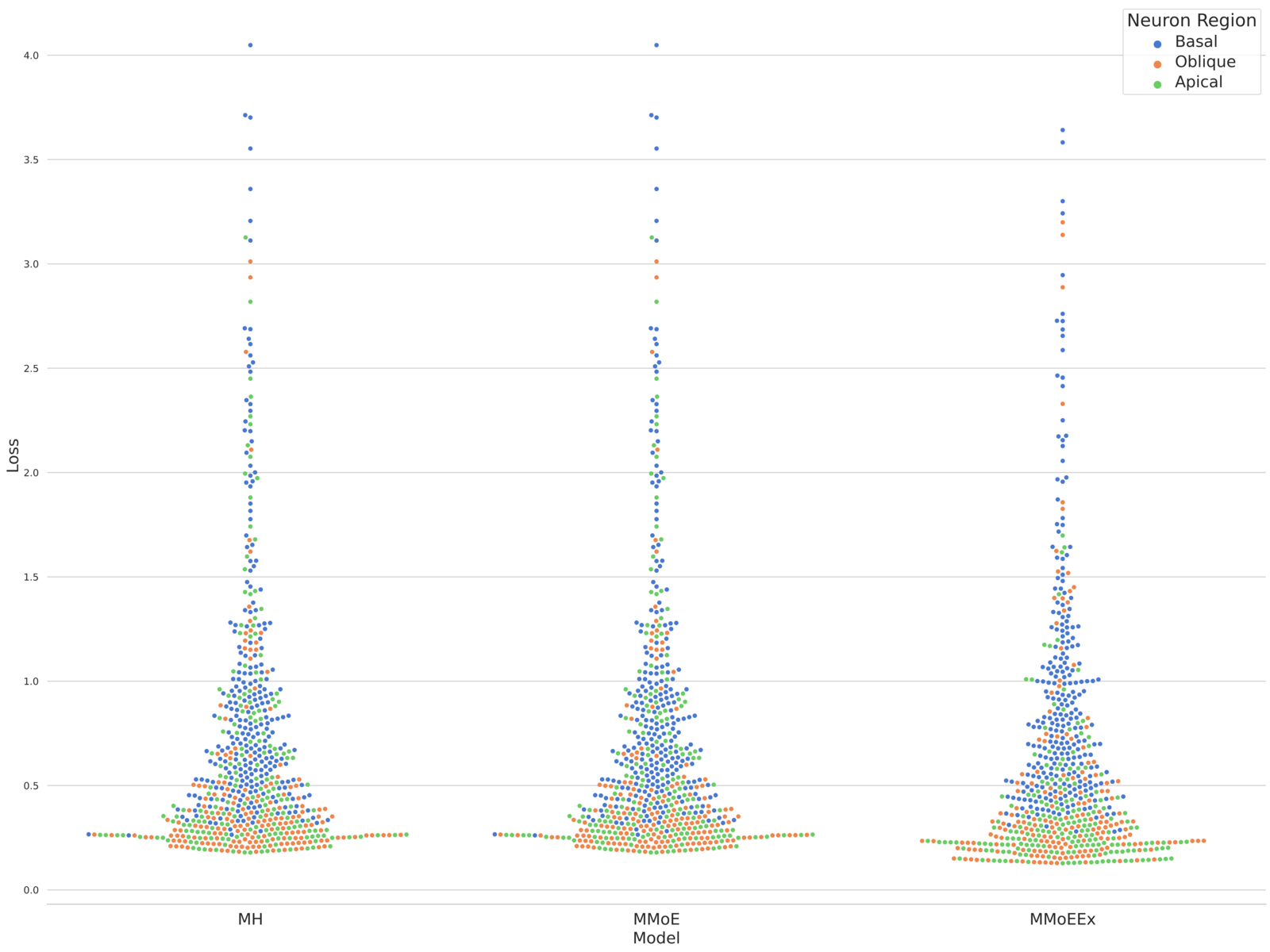

Supplement: S3 Fig — (TIFF) [file pcbi.1011728.s004.tiff]

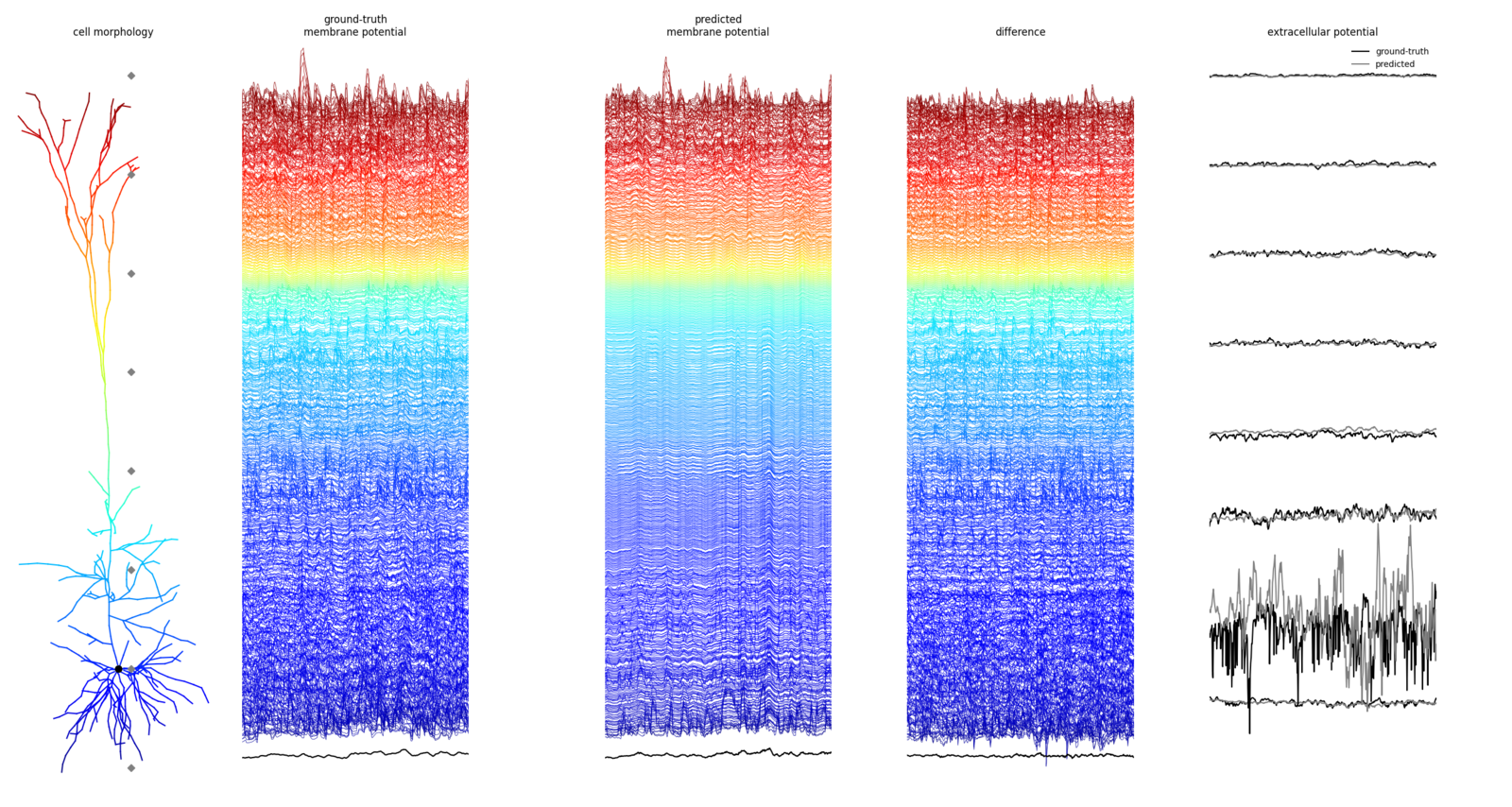

Supplement: S4 Fig — Illustration of a biophysically-detailed model of a multi-compartment cortical layer V pyramidal cell model. Membrane voltages as calculated by a biophysically-detailed simulations of the multi-compartment model, used as the ground truth throughout this paper. Membrane voltages as predicted by our best-performing multi-task learning architecture, one time step at a time (1 ms). Comparison between the ground truth and predicted extracellular potentials calculated at eight points representing the position of the electrodes. (TIFF) [file pcbi.1011728.s005.tiff]
